# DDEC: Dragon database of genes implicated in esophageal cancer

**DOI:** 10.1186/1471-2407-9-219

**Published:** 2009-07-06

**Authors:** Magbubah Essack, Aleksandar Radovanovic, Ulf Schaefer, Sebastian Schmeier, Sundararajan V Seshadri, Alan Christoffels, Mandeep Kaur, Vladimir B Bajic

**Affiliations:** 1 South African National Bioinformatics Institute, University of the Western Cape, Bellville, South Africa; 2 Computational Bioscience Research Center (CBRC), Mathematical and Computer Science and Engineering Division, King Abdullah University of Science and Technology (KAUST), Thuwal, Saudi Arabia

## Abstract

**Background:**

Esophageal cancer ranks eighth in order of cancer occurrence. Its lethality primarily stems from inability to detect the disease during the early organ-confined stage and the lack of effective therapies for advanced-stage disease. Moreover, the understanding of molecular processes involved in esophageal cancer is not complete, hampering the development of efficient diagnostics and therapy. Efforts made by the scientific community to improve the survival rate of esophageal cancer have resulted in a wealth of scattered information that is difficult to find and not easily amendable to data-mining. To reduce this gap and to complement available cancer related bioinformatic resources, we have developed a comprehensive database (Dragon Database of Genes Implicated in Esophageal Cancer) with esophageal cancer related information, as an integrated knowledge database aimed at representing a gateway to esophageal cancer related data.

**Description:**

Manually curated 529 genes differentially expressed in EC are contained in the database. We extracted and analyzed the promoter regions of these genes and complemented gene-related information with transcription factors that potentially control them. We further, precompiled text-mined and data-mined reports about each of these genes to allow for easy exploration of information about associations of EC-implicated genes with other human genes and proteins, metabolites and enzymes, toxins, chemicals with pharmacological effects, disease concepts and human anatomy. The resulting database, DDEC, has a useful feature to display potential associations that are rarely reported and thus difficult to identify. Moreover, DDEC enables inspection of potentially new 'association hypotheses' generated based on the precompiled reports.

**Conclusion:**

We hope that this resource will serve as a useful complement to the existing public resources and as a good starting point for researchers and physicians interested in EC genetics. DDEC is freely accessible to academic and non-profit users at http://apps.sanbi.ac.za/ddec/. DDEC will be updated twice a year.

## Background

The major histological form of esophageal cancer (EC), esophageal squamous cell carcinoma (ESCC), comprises 90% of ECs worldwide [1,2]. The poor prognosis of EC results in a five year survival rate of 5–20% [3]. The lethality of EC stems from our inability to detect the disease during the early stage, combined with the lack of effective therapies for advanced-stage disease. Like most diseases, EC arises as a consequence of errors occurring in the cellular regulatory system or errors being introduced into the genome as mutations causing cellular behavior to deviate from the norm [4]. Identifying the mechanisms by which the genomic information is controlled in EC will provide further insights into partially understood cellular and molecular functioning that characterizes this disease.

Gene expression in EC is a multifunctional process influenced by chromatin remodeling and the interplay between transcription regulatory proteins and DNA sequences known as transcription factor binding sites (TFBSs) [5,6]. This combination of transcription regulatory proteins, TFBSs, and affected transcripts, defines the transcription regulatory networks (TRNs) that are responsible for the regulation of every transcript encoded in the genome. Knowledge of these transcripts and the control mechanisms of their initiation set the stage for inferring transcriptional regulatory networks and may help in search for the therapeutic mechanisms to potentially correct or compensate for the errors underlying pathological states of EC.

Efforts made by the scientific community to improve the survival rate associated with EC have resulted in a wealth of scattered research data. Researchers need to sieve through this scattered research data to identify relevant research findings. However, this phase hampers the research process as the compiling of the relevant information is tedious and time consuming. In an attempt to enhance research endeavors related to EC we have developed Dragon Database of Genes Implicated in Esophageal Cancer (DDEC) as an integrated knowledge database that contains information about various genes differentially expressed in EC. It should be noted that there are two initiatives aimed at coordinating activities in producing resources related to cancer research, such as the International Cancer Genome Consortium – ICGC http://www.icgc.org/ and caBIG (cancer Biomedical Informatics Grid™, http://cabig.cancer.gov/. These two intend to promote specific data formats and other conditions that will enable easier integration of cancer-related resources. There are cancer related databases that include information on EC, such as Cancer Gene Expression Database (CGED) [7], PDQ [8] and Oncomine [9]. CGED houses a collection of gene expression and clinical data from a large number of patients with major cancers including EC. CGED expression data have been obtained by adaptor-tagged competitive PCR (ATAC-PCR) and allows researchers to explore the correlation between gene expression and clinical data for future diagnostic application [7]. PDQ is the National Cancer Institute's (NCI's) cancer database that includes peer-reviewed summaries on cancer treatment, screening, prevention, genetics, and complementary and alternative medicine [8]. The Oncomine initiative collects and analyzes all published cancer microarray data and currently house EC-related microarray data [9]. However, none of the current public databases focuses on genes implicated in EC and their potential associations with other relevant biological, biochemical and medical entities. Moreover, DDEC provides a combination of features for exploration of information related to EC-implicated genes that cannot be found elsewhere, such as filtering for putative transcription factors shared amongst promoters of EC-implicated genes, inference of association networks and precompiled reports that provide insights into other human genes and proteins, metabolites and enzymes, toxins, chemicals with pharmacological effects, disease concepts and human anatomy associated with differentially expressed EC-implicated genes. It also enables finding rare information that will be likely missed in the common literature search. As a special feature, DDEC provides a module for generation of 'association hypotheses' between concepts related to EC-implicated genes. Batch queries and database dump are also provided. We thus believe that DDEC represents a useful complement to the existing databases and will contribute to more efficient EC-related research. DDEC is freely accessible for academic and non-profit users at http://apps.sanbi.ac.za/ddec/. The semi-automated methodology used to populate DDEC genes and related data will be used to update the database twice a year.

## Construction and content

**Figure 1:**
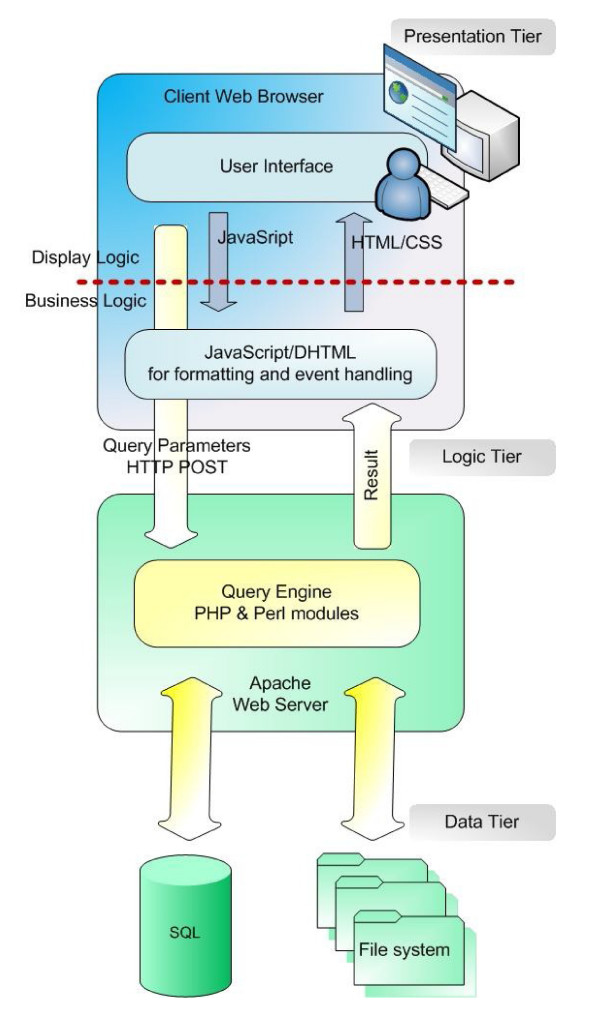
**The schematic representation of the DDEC structure**. The DDEC is based on the three-tier (layer) architecture, namely; data, logic, and presentation.

The DDEC is based on the three-tier (layer) (data, logic and presentation) architecture (Figure 1). The presentation layer is web-based and implemented in DHTML and Javascript. The logic layer was implemented as a number of server side PHP and Perl modules interfaced with the data layer. Data layer is MySQL, and for the text-mining purposes, file system based. The relational database design strictly distinguishes between tables that contain data entities and tables that establish logical connections between these data entities. The central data entity is the gene, to which most other data entities are linked. Other important data entities are transcription related such as transcription start sites (TSSs) and transcription factors (TFs). This is reflected in the entry points that a user can chose between on the top level of the web-interface.

Information in the DDEC is structured into four distinct parts:

(I) Platform that can be used to search the integrated gene information through standardized vocabularies.

(II) Selection of the genes of interest from the list. This search criteria provides users with gene details such as; general information, gene in other resources, experimental evidence, related proteins, associated pathways, associated diseases, orthologous genes, regulations and text-mined reports that can support building interactive association networks.

(III) Transcription regulation information which includes all putative TFBSs for the EC-implicated genes in DDEC. This segment is useful for gene regulation studies since TFBSs of interest can be selected and the results will list each TFBS and gene promoter with corresponding TFBSs. Genes sharing all the selected TFBSs are listed as well.

(IV) Batch queries and data download interface is provided to increase utility for users.

DDEC contains information on EC-implicated genes compiled based on scientific publications from PubMed. The PubMed database was queried with keyword expression: "esophageal (cancer OR cancers OR tumor OR tumors OR carcino* OR adenocarc* OR malign* OR neoplasm*)" on 31/01/2008 and 35,892 PubMed abstracts were retrieved. The search for relevant publications was further refined using the licensed Dragon Exploration System (DES) from OrionCell http://www.orioncell.org, that has an integrated Biomedical Text-Miner tool. DES retrieved a list of 1677 putative genes associated with EC from the extracted abstracts. Biologists then evaluated information about experimental conditions these genes have been subjected to using full-text articles whenever possible, and abstracts in other cases. When the available information was insufficient to deduce the correct experimental conditions, the gene has been discarded. Taking into account that experimental conditions influence gene expression, DDEC provide details of the cell line, tissue or cell type, expression status, disease stage, tumor grade, esophageal cancer type and laboratory method reported in literature.

A final list of 529 genes was identified in this way and used to populate the database. The general information about the genes, which include HGNC ID, approved symbol, approved name, entrez ID, previous symbol, previous name, aliases, OMIM-related information, and chromosome location, were extracted from sources such as HUGO [10]http://www.genenames.org/ and GeneCards [11]http://www.genecards.org/index.shtml. Included in the database are gene related identifiers such as EMBL [12]http://www.ebi.ac.uk/embl/, Ensembl [13]http://www.ensembl.org/index.html, Refseq [14], Genbank [15]http://www.ncbi.nlm.nih.gov/, Unigene [16]http://www.ncbi.nlm.nih.gov/sites/entrez?db=unigene&orig_db, Uniprot [17]http://www.ebi.ac.uk/uniprot/, Swiss-Prot [18]http://www.expasy.ch/sprot/ and PDB [19]http://www.rcsb.org/pdb/home/home.do. ID conversion tools like IDconvertor [20]http://idconverter.bioinfo.cnio.es/ and Onto-tools [21]http://vortex.cs.wayne.edu/ontoexpress/servlet/UserInfo were used to convert between different types of identifiers. A summary of the statistics of the above mentioned features are listed in documentation. We have provided links to the relevant sources of data such as gene ontologies [22]http://www.geneontology.org/, Evoc [23]http://www.evocontology.org/, and Reactome pathway data [24]http://www.reactome.org/.

As a useful feature, we generated lists of putative TFBSs that map to the promoter regions of EC-implicated genes allowing users to identify genes that share common TFBSs. For this purpose, promoter sequences were extracted using mainly FANTOM3 CAGE tag data [25], as well as TOUCAN v. 3.0.2 [26]. To map TFBSs to promoters we used the TRANSFAC Professional database v.11.4 [27]. All TRANSFAC mammalian matrix models of binding sites [28]were mapped using the Match™ program with *minFP *profiles for optimized thresholds of the matrix models [29]. The complete list of 529 genes was used to extract promoter sequences for the identification of putative TFBSs. Promoter sequences of 409 genes (1200 bp upstream and 200 bp downstream from the transcription start site, TSS) were extracted from the Fantom3 CAGE tag data that correspond to 1582 transcription start sites (TSSs) that each has at least five tags in the tag cluster and a minimum of three tags in the representative tag [25]. An additional 108 promoter sequences (1200 bp upstream and 200 bp downstream from the TSS) were extracted using Toucan v. 3.0.2 [26].

As an additional feature, for each of the 529 EC-implicated genes, we extracted all related PubMed documents and analyzed them using DES. DES uses a dictionary based text-mining approach to extract information used for the precompiled reports by mapping the entities from the dictionaries to the submitted PubMed documents. We applied six manually curated DES dictionaries namely; human genes and proteins, metabolites and enzymes, toxins, chemicals with pharmacological effects, disease concepts and human anatomy. These dictionaries were compiled from literature and public databases. The accuracy of this integrated data has been evaluated in Sagar *et al*. in terms of precision, recall and F-measure. The analysis of the results displayed precision and recall ranging from 81%–100% and with an average F-measure of 92.9% for the *SCN1A *gene [30]. The precompiled reports in this study are incorporated in the DDEC and provide the user with a possibility to inspect possible interactions associated with the genes of interest and associated networks of relevant biomedical entities. An additional feature in DES allows for hypotheses to be generated between two dictionary entries that are linked to a common dictionary entry. This tool allows the user to test the hypotheses generated by retrieving PubMed documents related to the two dictionary terms linked through the hypothesis, if no PubMed documents are retrieved the hypothesis may warrant further exploration. This functioning of the text-mining modules of DDEC is based on similar concepts as used in Pan *et al*. [31] and Bajic *et al*. [32]. DES has also been employed in the creation of a module for the ovarian cancer database, DDOC [33].

Batch queries and data download are provided to increase utility for users. Further, a database dump has been provided to support integration with other database resources.

The above outlined process of biocurated data collection and integration will be repeated twice yearly as an update process. Updates will incorporate extracting abstracts from the last update day to current day. This semi-automated process is more time consuming than current automated update systems but has the advantage of reducing redundant information.

## Utility and discussion

DDEC provides a comprehensive compilation of information obtained from published EC research, complemented with the information from public databases and information derived from computational analysis. The information captured in DDEC is centered on genes differentially expressed in EC. The information used for selection of genes to be included in DDEC was curated by biologists. Only genes that satisfy all conditions listed below are included in DDEC:

(i) Genes that are differentially expressed in human EC with experimental proof.

(ii) Differential expression of EC-implicated genes has not been influenced by anti-cancer therapy.

(iii) Differentially expressed EC-implicated genes have not been artificially constructed.

Microarray data has been excluded at this stage as the results obtained using high throughput technologies are debatable in terms of deciding about a meaningful level of gene expression and statistical methods used for analysis and interpretation of data [34,35]. However, as a future prospect we will expand the database by adding a subset for raw expression data and analysis of the EC-related microarray data.

DDEC contains precompiled text-mined and data-mined reports that allow for easy exploration of information about associations of EC-implicated genes with other genes and proteins, metabolites and enzymes, toxins, chemicals with pharmacological effects, disease concepts, human anatomy, pathways and pathway reactions. Moreover, DDEC provides for potentially new 'association hypotheses' generated in the precompiled reports. It also provides frequency of associations that allows users to observe rare associations with the genes of interest that will usually be overlooked in a normal literature search taking into account the huge volume of data available. DDEC can be used to answer questions such as:

(1) Is my gene of interest differentially expressed in EC, i.e. is it an EC-implicated gene as defined here?

(2) Which putative transcription factors regulate the expression of an EC-implicated gene or sets of these genes?

(3) Which of the other EC-implicated genes in DDEC are regulated by the same transcription factor (or factors) as the gene of interest?

(4) My gene of interest has putative associations with other biomedical concepts. What are these concepts and what are the documents from which such associations are deduced so that I can explore them?

The potential uses and advantages of the database are described in the documentation section http://apps.sanbi.ac.za/ddec/ddec.pdf. An example of data analysis has been included in the documentation and should help users to understand and utilize different functions implemented in this database to maximize information exploration and extraction.

Kaur *et al*. recently published DDOC, an ovarian cancer (OC) database housing 379 OC-related genes using the same database model and query interface [33]. To explore whether the EC and OC database content characterize functionally distinct groups of genes, the categories where probed for statistical over-representation of GO terms [22,36]. For this analysis we compared the EC and OC gene lists. We found 123 genes to be common to both cancer types while 406 genes were unique to EC and 256 genes were unique to OC. Generally, all categories were characterized by the majority of genes forming part of the broad terms, apoptosis and cell cycle. However, these categories were primarily over-represented for the genes common to both EC and OC (see Table 1). The gene list unique to EC was found to be enriched in functionally distinct groups such as 'neuron differentiation and development' and 'epidermis development' while the gene list unique to OC was found to be enriched in functionally distinct groups such as 'sex differentiation and development' and 'embryonic development' (see Table 1).

**Table 1:** A comparison of the DDEC and DDOC gene lists.

Gene Ontology terms representing functionally distinct groups	Genes unique for Esophageal Cancer (EC)	Gene unique for Ovarian Cancer (OC)	Genes common to EC and OC
Neuron differentiation and development	3.03	0.77	0

Epidermis development	5.91	1.06	1.66

Sex differentiation and development	0.36	1.36	0

Embryonic development	0.76	1.85	0

Regulation of apoptosis	8.09	9.84	13.22

Regulation of cell cycle	11.99	11.31	14.73

* The values tabulated in Table 1 represent the overall enrichment score for the group based on EASE scores of each term members. The higher the score the more enriched.

A comparison of Esophageal and Ovarian Cancer genes by characterizing functionally distinct groups based on Gene Ontology terms.

We further identified which KEGG pathways (see additional file 1) are enriched for the genes unique to EC, genes unique to OC and the genes common to EC and OC [37]. We found the MAPK signaling pathway, ErbB signaling pathway and p53 signaling pathway to be most pronounced pathways for genes common to EC and OC. The pathways most pronounced for the genes unique to EC were the MAPK signaling pathway, Wnt signaling pathway, with androgen and estrogen metabolism being unique to this group. The MAPK signaling pathway, ErbB signaling pathway and TGF-beta signaling pathways were most pronounced for the genes unique to OC.

Above analysis suggests that distinct categories of genes participating in specific pathways are involved in pathogenesis of different types of cancers. These cancer specific categories of genes can be investigated as potential biomarkers for prognosis and diagnosis of the disease.

In future, we intend to incorporate the effect of current therapeutic drugs. Additional features that may enhance search and retrieval of DDEC information will be added in due course, as well as incorporation of DDEC into ICGC, caBIG and LinkOut. DDEC will further be updated twice a year and will continue to grow in both content and functionality.

## Conclusion

DDEC is an integrated knowledge database aimed at representing a gateway to EC-related data. DDEC houses information associated with 529 hand-curated human genes implicated in EC and allows the users to easily access the wealth of EC related data that is typically difficult to find and not easily amendable to data mining. Users are also provided with the DES interface that allows for the easy exploration of information, viewing of potential associations that are rarely reported and thus difficult to identify and inspection of potentially new 'association hypotheses' generated based on the precompiled reports. We hope that this resource will serve as a useful complement to the existing public resources and as a good starting point for researchers and physicians interested in EC genetics.

## Availability and requirements

DDEC is freely accessible to academic and non-profit users at http://apps.sanbi.ac.za/ddec/.

## Competing interests

Vladimir B. Bajic and Aleksandar Radovanovic are partners in the OrionCell company whose product, Dragon Exploration System (DES), has been used in creation of DDEC precompiled reports. Other authors declare no conflict of interest.

## Authors' contributions

ME, MK and VBB conceptualized the study, analyzed data and wrote the manuscript. AR, US, SVS, SS and AC developed the database. AR and VBB developed the DES system. All authors read and approved the final manuscript.

## Pre-publication history

The pre-publication history for this paper can be accessed here:

http://www.biomedcentral.com/1471-2407/9/219/prepub

## Supplementary Material

Additional file 1 **The implication of esophageal and ovarian cancer genes in KEGG pathways**. This additional file is subdivided into three worksheets that list the genes common to EC and OC, genes unique to EC and genes unique to OC. Each worksheet further lists the gene name of each entry, the associated Entrez Gene ID and KEGG pathways. Click here for file

## References

[B1] Stoner GD Rustgi AK Biology of the esophageal squamous cell carcinoma Gastrointest Cancers Biol Diagn 1995 8 141 146

[B2] WHO The World Health Report 1997 – conquering suffering, enriching humanity World Health Forum 1997 18 248 260 9478137

[B3] Reed CE Surgical management of esophageal carcinoma Oncologist 1999 4 95 105 10337379

[B4] De LL Curia MC Aceto GM Toracchio S Colucci G Russo A Mariani-Constantini R Cama A Analysis of extended genomic rearrangements in oncological research Ann Oncol 2007 18 Suppl 6 vi173 vi178 17591817 10.1093/annonc/mdm251

[B5] Gilbert N Gilchrist S Bickmore WA Chromatin organization in the mammalian nucleus Int Rev Cytol 2005 242 283 336 10.1016/S0074-7696(04)42007-5 15598472

[B6] Cremer T Cremer C Chromosome territories, nuclear architecture and gene regulation in mammalian cells Nat Rev Genet 2001 2 292 301 10.1038/35066075 11283701

[B7] Kato K Yamashita R Matoba R Monden M Noguchi S Takagi T Nakai K Cancer gene expression database (CGED): a database for gene expression profiling with accompanying clinical information of human cancer tissues Nucleic Acids Res 2005 33 D533 D536 15608255 10.1093/nar/gki117 PMC540071

[B8] Thiemann KM Frost MH Thompson RA A multifaceted educational approach to increasing awareness and use of physician data query (PDQ) J Cancer Educ 1999 14 78 82 10397481 10.1080/08858199909528584

[B9] Rhodes DR Yu J Shanker K Deshpande N Varambally R Ghosh D Barrette T Pandey A Chinnaiyan AM ONCOMINE: a cancer microarray database and integrated data-mining platform Neoplasia 2004 6 1 6 15068665 10.1016/s1476-5586(04)80047-2 PMC1635162

[B10] Eyre TA Ducluzeau F Sneddon TP Povey S Bruford EA Lush MJ The HUGO Gene Nomenclature Database, 2006 updates Nucleic Acids Res 2006 34 D319 D321 16381876 10.1093/nar/gkj147 PMC1347509

[B11] Safran M Solomon I Shmueli O Lapidot M Shen-Orr S Adato A Ben-Dor U Esterman N Rosen N Peter I Olender T Chalifa-Caspi V Lancet D GeneCards 2002: towards a complete, object-oriented, human gene compendium Bioinformatics 2002 18 1542 1543 10.1093/bioinformatics/18.11.1542 12424129

[B12] Kulikova T Akhtar R Aldebert P Althorpe N Andersson M Baldwin A Bates K Bhattacharyya S Bower L Browne P EMBL Nucleotide Sequence Database in 2006 Nucleic Acids Res 2007 35 D16 D20 17148479 10.1093/nar/gkl913 PMC1897316

[B13] Flicek P Aken BL Beal K Ballester B Caccamo M Chen Y Clarke L Coates G Cunningham F Cutts T Ensembl 2008 Nucleic Acids Res 2008 36 D707 D714 18000006 10.1093/nar/gkm988 PMC2238821

[B14] Pruitt KD Tatusova T Maglott DR NCBI reference sequences (RefSeq): a curated non-redundant sequence database of genomes, transcripts and proteins Nucleic Acids Res 2007 35 D61 D65 17130148 10.1093/nar/gkl842 PMC1716718

[B15] Benson DA Karsch-Mizrachi I Lipman DJ Ostell J Sayers EW GenBank Nucleic Acids Res 2009 37 D26 D31 18940867 10.1093/nar/gkn723 PMC2686462

[B16] Wheeler DL Barrett T Benson DA Bryant SH Canese K Chetvernin V Church DM Dicuccio M Edgar R Federhen S Database resources of the National Center for Biotechnology Information Nucleic Acids Res 2008 36 D13 D21 18045790 10.1093/nar/gkm1000 PMC2238880

[B17] UniProt Consortium The universal protein resource (UniProt) Nucleic Acids Res 2008 36 D190 D195 18045787 10.1093/nar/gkm895 PMC2238893

[B18] Gasteiger E Jung E Bairoch A SWISS-PROT: connecting biomolecular knowledge via a protein database Curr Issues Mol Biol 2001 3 47 55 11488411

[B19] Berman H Henrick K Nakamura H Markley JL The worldwide Protein Data Bank (wwPDB): ensuring a single, uniform archive of PDB data Nucleic Acids Res 2007 35 D301 D303 17142228 10.1093/nar/gkl971 PMC1669775

[B20] Alibes A Yankilevich P Canada A az-Uriarte R ID converter and IDClight: conversion and annotation of gene and protein IDs BMC Bioinformatics 2007 8 9 17214880 10.1186/1471-2105-8-9 PMC1779800

[B21] Khatri P Desai V Tarca AL Sellamuthu S Wildman DE Romero R Draghici S New Onto-Tools: Promoter-Express, nsSNPCounter and Onto-Translate Nucleic Acids Res 2006 34 W626 W631 16845086 10.1093/nar/gkl213 PMC1538776

[B22] Ashburner M Ball CA Blake JA Botstein D Butler H Cherry JM Davis AP Dolinski K Dwight SS Eppig JT Gene ontology: tool for the unification of biology. The Gene Ontology Consortium Nat Genet 2000 25 25 29 10.1038/75556 10802651 PMC3037419

[B23] Kelso J Visagie J Theiler G Christoffels A Bardien S Smedley D Otgaar D Greyling G Jongeneel CV McCarthy MI eVOC: a controlled vocabulary for unifying gene expression data Genome Res 2003 13 1222 1230 12799354 10.1101/gr.985203 PMC403650

[B24] Vastrik I D'Eustachio P Schmidt E Gopinath G Croft D de Bono B Gillespie M Jassal B Lewis S Matthews L Reactome: a knowledge base of biologic pathways and processes Genome Biol 2007 8 R39 17367534 10.1186/gb-2007-8-3-r39 PMC1868929

[B25] Carninci P Sandelin A Lenhard B Katayama S Shimokawa K Ponjavic J Semple CA Taylor MS Engstrom PG Frith MC Genome-wide analysis of mammalian promoter architecture and evolution Nat Genet 2006 38 626 635 10.1038/ng1789 16645617

[B26] Aerts S Van LP Thijs G Mayer H de MR Moreau Y De Moor B TOUCAN 2: the all-inclusive open source workbench for regulatory sequence analysis Nucleic Acids Res 2005 33 W393 W396 15980497 10.1093/nar/gki354 PMC1160115

[B27] Wingender E Chen X Fricke E Geffers R Hehl R Liebich I Krull M Matys V Michael H Ohnhauser R The TRANSFAC system on gene expression regulation Nucleic Acids Res 2001 29 281 283 11125113 10.1093/nar/29.1.281 PMC29801

[B28] Matys V Kel-Margoulis OV Fricke E Liebich I Land S Barre-Dirrie A Reuter I Chekmenev B Krull M Hornischer K TRANSFAC and its module TRANSCompel: transcriptional gene regulation in eukaryotes Nucleic Acids Res 2006 34 D108 D110 16381825 10.1093/nar/gkj143 PMC1347505

[B29] Kel AE Gossling E Reuter I Cheremushkin E Kel-Margoulis OV Wingender E MATCH: A tool for searching transcription factor binding sites in DNA sequences Nucleic Acids Res 2003 31 3576 3579 12824369 10.1093/nar/gkg585 PMC169193

[B30] Sagar S Kaur M Dawe A Seshadri SV Christoffels A Schaefer U Radovanovic A Bajic VB DDESC: Dragon database for exploration of sodium channels in human BMC Genomics 2008 9 622 19099596 10.1186/1471-2164-9-622 PMC2631582

[B31] Pan H Zuo L Choudhary V Zhang Z Leow SH Chong FT Huang Y Ong VW Mohanty B Tan SL Dragon TF Association Miner: a system for exploring transcription factor associations through text-mining Nucleic Acids Res 2004 32 W230 W234 15215386 10.1093/nar/gkh484 PMC441622

[B32] Bajic VB Veronika M Veladandi PS Meka A Heng MW Rajaraman K Pan H Swarup S Dragon Plant Biology Explorer. A text-mining tool for integrating associations between genetic and biochemical entities with genome annotation and biochemical terms lists Plant Physiol 2005 138 1914 1925 16172098 10.1104/pp.105.060863 PMC1183383

[B33] Kaur M Radovanovic A Essack M Schaefer U Maqungo M Kibler T Schmeier S Christoffels A Narasimhan K Choolani M Bajic VB Database for exploration of functional context of genes implicated in ovarian cancer Nucleic Acids Res 2009 37 D820 D823 18790805 10.1093/nar/gkn593 PMC2686485

[B34] Smyth GK Yang YH Speed T Statistical issues in cDNA microarray data analysis Methods Mol Biol 2003 224 111 136 12710670 10.1385/1-59259-364-X:111

[B35] Pritchard CC Hsu L Delrow J Nelson PS Project normal: defining normal variance in mouse gene expression Proc Natl Acad Sci USA 2001 98 13266 13271 11698685 10.1073/pnas.221465998 PMC60859

[B36] Dennis G Jr Sherman BT Hosack DA Yang J Gao W Lane HC Lempicki RC DAVID: Database for Annotation, Visualization, and Integrated Discovery Genome Biol 2003 4 3 10.1186/gb-2003-4-5-p3 PMC193660 12734009

[B37] Goto S Bono H Ogata H Fujibuchi W Nishioka T Sato K Kanehisa M Organizing and computing metabolic pathway datain terms of binary relations Pac Symp Biocomput 1997 175 186 9390290

